# Clinical considerations in the management of hidradenitis suppurativa in women

**DOI:** 10.1016/j.ijwd.2021.10.012

**Published:** 2021-10-29

**Authors:** Emily K. Kozera, Michelle A. Lowes, Jennifer L. Hsiao, John W. Frew

**Affiliations:** a Liverpool Hospital Department of Dermatology, Sydney, Australia; b Rockefeller University, New York, New York; c Division of Dermatology, University of California, Los Angeles, California; d University of New South Wales, Sydney, Australia

**Keywords:** Hidradenitis suppurativa, estrogen, progesterone, hormones, women, childbirth, breastfeeding, menopause, quality of life, psychosexual function

## Abstract

Hidradenitis suppurativa (HS) is a chronic, inflammatory disease of the skin with a predilection for women. The role of sex hormones, including estrogen and progesterone, is incompletely understood, but alterations in hormone levels may play a role in disease activity for many patients. Specific clinical considerations should be made for women with HS, particularly in the setting of pregnancy, childbirth, breastfeeding, and menopause. Current knowledge gaps regarding HS include the cumulative impact of disease across an individual's lifespan, as well as the mechanistic role of sex hormones in the disease. An improved understanding of the pathophysiologic role of hormones in HS would optimize our ability to use targeted therapies for hormonally driven disease. Psychological and psychosexual support for women with HS is an important facet of any holistic management strategy for the disease. This article integrates up-to-date pathogenic and mechanistic insights with evidence-based clinical management to optimize care for women with HS.

**What is known about this subject in regard to women and their families?**
• Hidradenitis suppurativa is a chronic, debilitating, inflammatory disorder with a propensity for women.
• Hormones appear to play a strong role in disease activity for many women.

**What is new from this article as messages for women and their families?**
• The mechanisms of how hormones influence disease activity in women with hidradenitis suppurativa (HS) is an area that needs further scientific investigation.
• Multidisciplinary management is needed to safely manage women with HS through pregnancy, childbirth, breastfeeding, and menopause.
• Psychological and psychosexual support for women with HS is an important facet of any holistic management strategy.

Alt-text: Unlabelled box

## Introduction

Hidradenitis suppurativa (HS) is a chronic skin disease with features of autoinflammation and autoimmunity, manifesting as painful nodules and abscesses, as well as malodorous, draining, epithelialized tunnels with a predilection for flexural areas of the skin. As a systemic inflammatory disorder, HS is associated with multiple comorbidities across organ systems, including inflammatory bowel disease, inflammatory arthropathy, as well as metabolic syndrome (Adelekun et al., 2020). In North American and European epidemiologic studies, HS has been found to disproportionately affect women of childbearing age (Adelkun et al, 2021; Garg et al., 2021; Miller et al., 2016). In the United States, an increased prevalence in African-American and biracial patients has been found. However, in global HS clinical trials, most participants have been Caucasian (Ding et al., 2021). Thus, available information regarding the role of hormones and mechanistic and translational studies in HS may not be entirely representative across ethnicities.

Women face unique disease burdens, including those associated with menstruation, hormonal fluctuation, sexual function, pregnancy, childbirth, and breastfeeding. This review article aims to discuss and present the mechanistic underpinnings regarding the role of sex hormones in the pathogenesis of HS in women, as well as discuss the published evidence and clinical management of HS in the setting of perimenstrual flares, pregnancy, childbirth, breastfeeding, and menopause.

## Hormones in pathogenesis of hidradenitis suppurativa

In recent years, there has been increasing observational, experimental, and therapeutic evidence that HS has features of a chronic, autoinflammatory, keratinization disorder (Frew, 2021). Hormone dysfunction is thought to play a role in the underlying pathogenesis given the association of HS with hormonal acne vulgaris, polycystic ovarian syndrome (PCOS), and fluctuations in HS disease severity associated with the menstrual cycle (Riis et al., 2016). The well-established links between PCOS, obesity, insulin resistance, and elevated levels of systemic proinflammatory mediators, such as insulin-like growth factor 1 (IGF-1), leukotrienes, and long chain fatty acids (Penno et al., 2020), provide theoretical mechanisms as to how hormones may influence disease activity in HS; however, mechanistic evidence in HS is incomplete. Links between androgen-receptor mediated inflammatory pathways and interleukin-23 have also been observed (Adelekun et al., 2021) and may present an alternate hypothesis on how the Th17 inflammatory pathway identified in HS is linked with hormonal dysregulation.

The current evidence for the effect of sex hormones on HS disease activity is largely based on an epidemiologic association, with a paucity of mechanistic data regarding how these hormones impact the inflammatory drive in the disease. Patients with HS have more than three times the crude prevalence of PCOS compared with patients without HS (Penno et al., 2020). Even among women with a diagnosis of HS who do not have PCOS, some women demonstrate clinical signs of androgen excess, including acne vulgaris, hirsutism, irregularities of the menstrual cycle, and infertility (Penno et al., 2020; Yan et al., 2018).

The existing HS pathogenic paradigm holds that end-organ (follicular) activity of sex hormones may play a role in disease pathogenesis (Frew, 2021). However, studies have not shown immunohistochemical evidence of dysregulated sex hormone receptors in the lesional skin of patients with HS when compared with healthy controls (Frew et al., 2018). Research in other fields has established that sex hormones have immune-modulating (both immune-activating and immune-suppressive) activity on dendritic cells, T-cell maturation, differentiation, and suppression of the Th1 immune response (Straub, 2014). The effect of sex hormones on the immune system differs between men and women and is influenced by the end organ, background cytokine milieu, and endogenous sex hormone levels (Straub, 2014; Xiao et al., 2016).

Estrogens in particular have immune-modulating mechanisms independent of canonical estrogen-response elements (Straub, 2014; Xiao et al., 2016). These pathways influence important immune pathways, such as nuclear factor kappa B (NF-κB), SP1, AP1, and phosphoinositide 3-kinase/Akt pathways associated with HS (Clark et al., 2017; Straub, 2014; Xiao et al., 2016). Estrogen metabolites (16-α estrogens) are known to modulate local immune responses in inflammatory arthritis and encourage insulin resistance. Both of these conditions are associated with HS, suggesting that 16-α estrogens may be mechanistically important in some patients.

With regard to progesterone and androgens, the nuclear localization of progesterone can influence proinflammatory transcription factors, including NF-kB, AP1, and nuclear factor of activated T cells. Given that 5-α reductase (important for conversion of testosterone to dihydrotestosterone) is IGF-1-dependent, there is conceivable interplay between IGF-1 and progesterone and androgen nuclear localization and signaling. Therefore, finasteride may have increased benefits in individuals with insulin resistance or diabetes, although this has not been systematically investigated.

Spironolactone is an aldosterone antagonist and has been associated with reduced inflammatory cytokine levels in various tissues (Zhang et al., 2014), as well as suppression of tumor necrosis factor-alpha, interleukin-6, and the inhibition of NF-κB phosphorylation and nitric oxide synthesis (Zhang et al., 2014). However, the precise mechanism through which spironolactone influences HS disease activity is not well described.

Despite a lack of mechanistic evidence, a clinical benefit is seen in patients with HS with antiandrogen therapy, including finasteride, spironolactone, and antiandrogenic progestogens (cyproterone acetate, chlormadinone acetate, drospirenone; Bereschchenko et al., 2018; Karagiannidis et al., 2016). Antiandrogen therapy is an important central tenet to the management of women with HS experiencing perimenstrual flares. Overall, further mechanistic work is needed to examine the pathways associated with inflammation in HS, specifically noncanonical signaling pathways and changes in gene transcription. The bimodal response between men and women (the same sex hormone having different inflammatory effects in men vs. women) suggests that the influence of hormones may be very different between the sexes and that sex-specific studies are needed to accurately dissect the role of sex hormones in HS.

## Perimenstrual hidradenitis suppurativa flares

Several studies have suggested perimenstrual flaring in HS. It has been reported that between 43% and 63% of women experience worsening of their HS around the time of menstruation (Collier et al, 2020a; Riis et al., 2016). Within this group of women who experience perimenstrual flaring, 78.9% reported that a flare in their HS would occur within the week preceding their menses, 18.9% experienced a flare during menses, and 2.3% experienced a flare after menses (Collier et al., 2020a). This study also found a significantly higher rate of menstrual acne flares in women with menstrual HS flares compared with those without menstrual HS flares, with 71.7% reporting a flare of their acne during the perimenstrual period (Collier et al., 2020a). These findings again suggest that hormonal influence is associated with HS flares.

In terms of management, limited evidence suggests that the combined oral contraceptive pill (combined low dose estrogen and progesterone) and spironolactone may aid in the management of perimenstrual flares in some women, although further mechanistic insights are needed to identify those women who will benefit most from hormonal therapy in this setting.

## Pregnancy and hidradenitis suppurativa

**Fig. 1:**
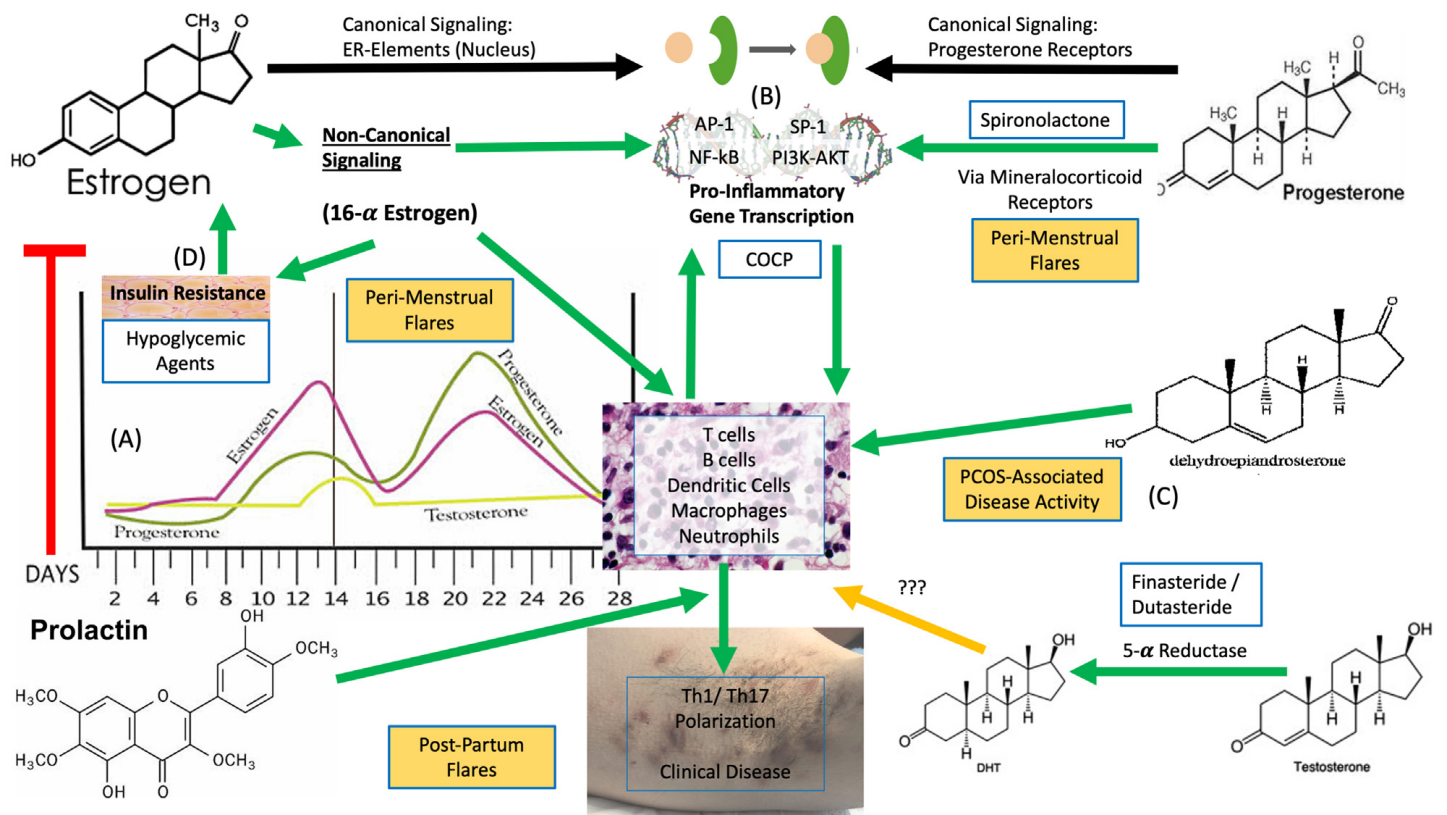
Potential mechanisms of hormones in hidradenitis suppurativa (HS), showing that (A) estrogen and progesterone naturally vary across the menstrual cycle, but (B) each of these sex hormones have sex-specific immune modulating actions, either by directly acting on transcription factors and gene expression in inflammatory cells or indirectly via incompletely understood mechanisms (via 16-α estrogen). This leads to TH1/TH17 polarization, which is well recognized as an inflammatory contributor to HS. The role of other sex hormones, including (C,D) dehydroepiandrosterone sulfate, insulin, and testosterone, is less completely understood in the setting of HS, and further mechanistic investigation is required. Red arrows indicate suppressive activity, yellow arrows indicate unknown activity, and green arrows indicate contributory activity.

A recent meta-analysis (Seivright et al., unpublished) found that, across 672 cases in eight studies published between 1986 and 2020, the rate of HS improvement during pregnancy was 24% and the rate of HS worsening was 20%. Across four studies of postpartum flare, 60% of patients reported a disease flare during the postpartum period. These data indicate a variable disease response to pregnancy. The relative relationship between estrogen and progesterone levels has been hypothesized to be associated with the degree of disease activity (Riis et al., 2016) given the dramatic reduction in estrogen and high prolactin levels associated with the postpartum period (Fig. 1).

Not only is pregnancy associated with disease fluctuations, including potential disease worsening, but some HS medications are either contraindicated during pregnancy or their safety has not been studied among pregnant women. Some common medications used in the management of HS that are contraindicated during pregnancy include oral tetracyclines, spironolactone, combined oral contraceptive pills, and retinoid therapies (Nesbitt et al., 2020; Perng et al., 2016). However, there are still many medications that can be used in pregnant patients with NS (Beltagy et al., 2021; Koh et al., 2019; Nesbitt et al., 2020), and patients must be counseled regarding this; a survey of 59 female patients with HS found that almost half believed that pregnancy necessitates stopping all HS medications for safety reasons (Adelekun et al., 2021).

Although more rigorous studies on biologic medications during pregnancy are needed, registry data are available for several biologic agents (Koh et al., 2019). The risks of utilizing a biologic agent during pregnancy must be weighed against the risk of uncontrolled severe HS disease. A survey of 49 HS specialists found that 59% reported that they have prescribed or continued use of biologics for pregnant patients with HS (Collier et al., 2020b). Commonly used antitumor necrosis factor-alpha biologics for HS, including adalimumab and infliximab, cross the placenta, so if continued until delivery, the patient's baby should not receive live vaccines until after 6 months of age. Some practitioners change to certolizumab during the third trimester for this reason because there is minimal placental transfer of certolizumab (Collier et al., 2020b; Mariette et al., 2017). However, data on efficacy of certolizumab in the treatment of HS are limited. Extrapolating from the rheumatology literature, a recent systematic review did not identify an increased risk of negative fetal outcomes during biologic therapy; however, HS-specific studies are required (Tsao et al., 2020).

It has been suggested that pregnant women with HS should also receive appropriate counseling with regard to appropriate weight gain during pregnancy (Fernandez et al., 2020). This is based on the association of increased body mass index with a risk of developing HS, although the exact mechanisms are incompletely understood. Traditional teaching states that increased mechanical stress on intertriginous areas leads to the retention of hair follicle materials, epidermal hyperplasia, and follicular rupture (Sakya et al., 2021). However, more recent research has suggested that other contributions, including the proinflammatory nature of adipose tissue and interactions between hormones and systemic inflammation, may play major roles in the pathophysiology of HS. One of the most commonly affected intertriginous sites affected during pregnancy is the abdominal pannus.

Women of childbearing age often inquire about the risk of their offspring developing HS. Genetic counseling for HS begins with interpreting patients’ medical history and education about inheritance of the disease in question. There are approximately 70 families with HS and autosomal dominant gamma-secretase mutations (*NCSTN, PSENEN*, and *PSEN1*; Wang et al., 2020), although the pathogenic relevance of these mutations is not yet clear (Frew et al., 2017). Patients with rare syndromic HS have mutations in certain genes in innate immune pathways, such as *MEFV* and *PSTPIP*1 (Vossen et al., 2018). A recent study in the Netherlands Twin Registry demonstrated that HS occurred in 1.2% of twins (Van Straalen et al., 2020). However, approximately 1 in 3 patients with HS describe a relative with HS (Van Straalen et al., 2020). Hence, patients of childbearing age with HS can be counseled that HS is currently understood to be due to a combination of factors including genetics, hormones, and other environmental factors. The risk of developing HS is higher if an individual has an affected parent; however, larger genetic studies are needed to provide more detailed data to patients with HS about the specific risks of HS.

**Fig. 2:**
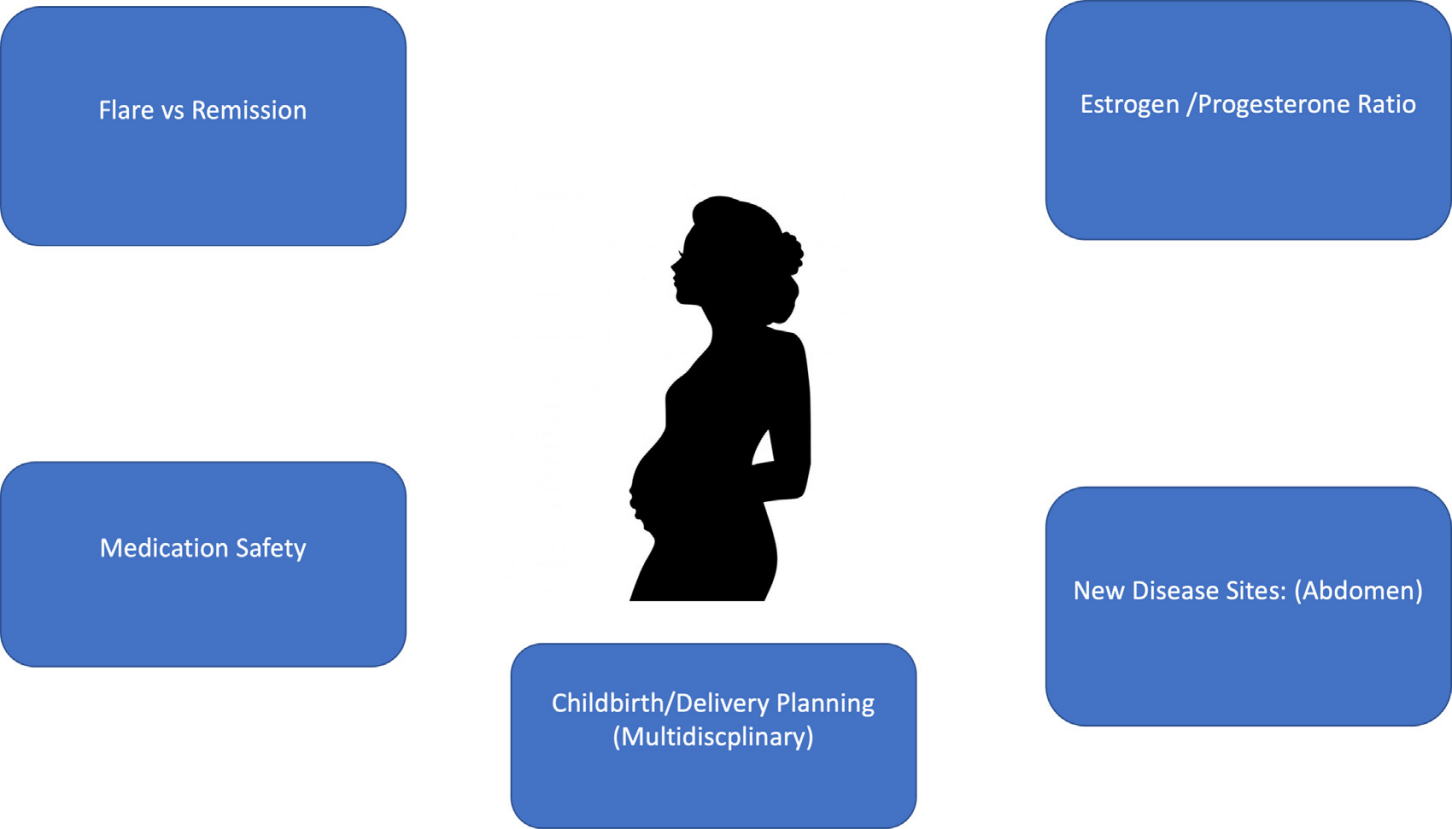
Pregnancy and hidradenitis suppurativa: Management issues.

Important management goals and clinical considerations in the antenatal period for women with HS are summarized in Figure 2. Continued close monitoring and management of HS disease during pregnancy is paramount to assess whether the HS will remit or flare during the course of the pregnancy. The balance between disease optimization for the mother and minimization of potential harm to the fetus should be considered. Intralesional steroids can be carefully given for disease flares. It is also vital that the woman is referred to various appropriate medical and allied health care teams. It has been widely demonstrated that integrated multidisciplinary management of patients with HS improves patient outcomes and satisfaction with care (Fernandez et al., 2020; Touhouche et al., 2020).

## Childbirth and hidradenitis suppurativa

Childbirth presents unique challenges for patients with HS. Fernandez et al. (2020) found that 3.1% of patients with anogenital HS who delivered vaginally reported that their HS interfered with vaginal delivery, and 23.5% believed vaginal delivery caused an HS flare. Of patients who reported having a Cesarean section delivery, 33.9% reported impaired incision healing secondary to HS, and 51.2% reported the development of new HS inflammatory nodules within their Cesarean section scar (Fernandez et al., 2020). A similar case is described by Darch et al. (2020), who reported on a 33-year-old female patient with HS who was managed with adalimumab until the end of the second trimester. The patient gave birth via Cesarean section and within 2 months after delivery developed multiple inflammatory nodules and tracts around her Cesarean scar (Darch et al., 2020).

Women with HS also have significantly lower odds (52.0%) of having a live birth compared with women without HS (70.74%; Sakya et al., 2021). Furthermore, women with HS are reportedly 2.51 times more likely to have an elective termination during pregnancy compared with women without HS (Sakya et al., 2021). There is also a case report in the literature of a miscarriage secondary to HS complications. A 40-year-old, 16-week pregnant patient with primarily vulvar HS presented to a hospital with large polypoidal lymphoedematous vulvar masses, malodourous and purulent discharge, and cellulitis of the groin. Within 24 hours of her presentation to doctors, the woman had a miscarriage secondary to ascending *Peptostreptococcus anaerobius* and group B-streptococcal bacterial infection (Ghamdi, 2020). This example demonstrates a rare but tragic consequence of severely infected vulvar HS.

**Fig. 3:**
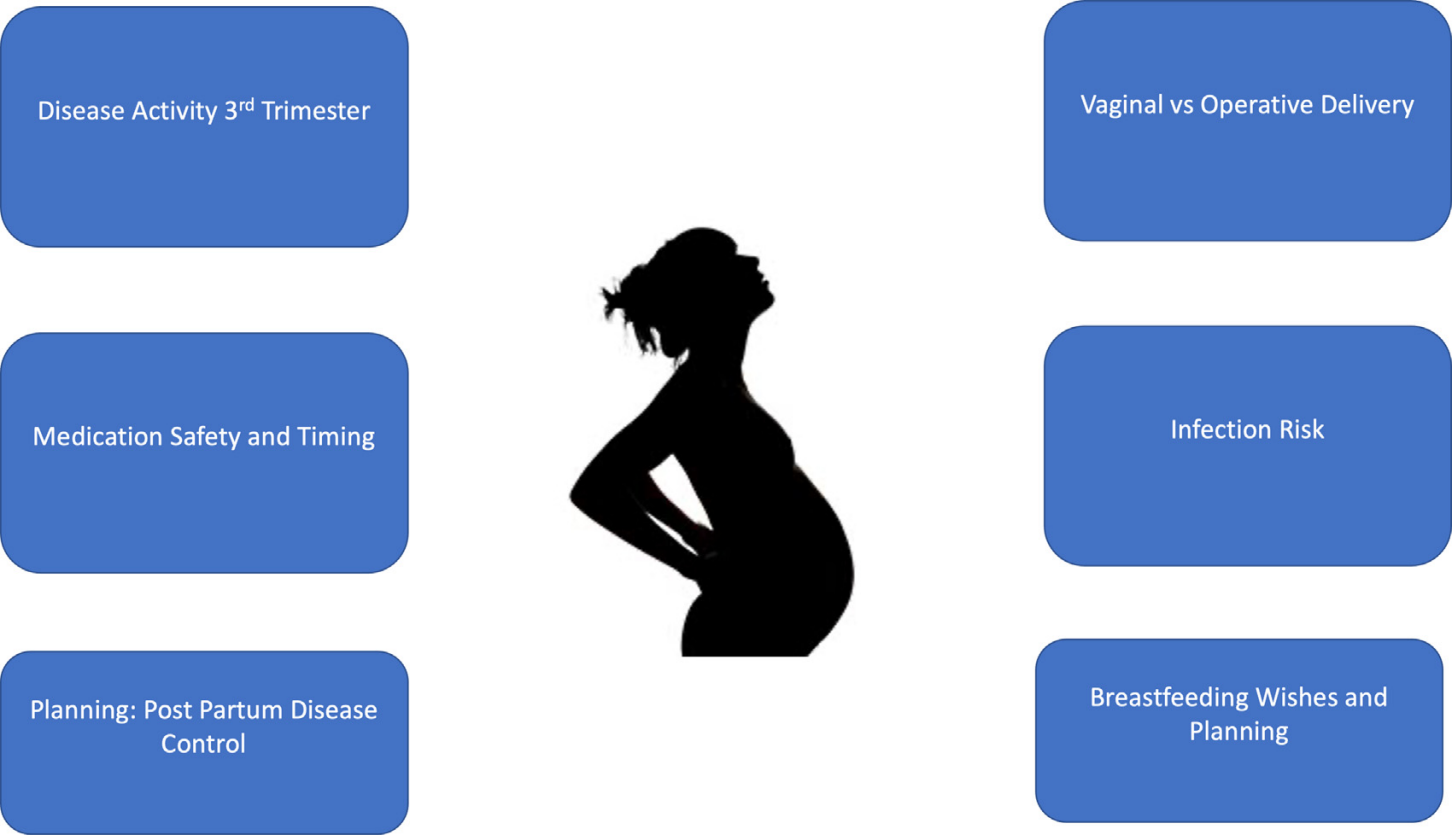
Childbirth and hidradenitis suppurativa: Management issues.

Important management goals and clinical considerations during the childbirth period for women with HS include optimizing disease control for the mother without causing harm to the fetus, discussing and choosing the most appropriate delivery method in conjunction with the obstetrician, and reducing the risk of secondary infection and related consequential sequelae (Fig. 3).

## Breastfeeding and hidradenitis suppurativa

Having HS lesions on the breasts may present a significant obstacle to breastfeeding. If a woman would like to pursue breastfeeding, the treatment of these lesions during pregnancy with localized therapies, such as intralesional corticosteroids or systemic medications (e.g., oral antibiotics), may make breastfeeding a more feasible option (Collier et al., 2020c).

Women with HS who are breastfeeding have limited pharmacological treatment options compared with those who are not breastfeeding. Topical therapies, including chlorhexidine wash, benzoyl peroxide, and topical clindamycin, are generally considered safe in breastfeeding women (Collier et al., 2021). Systemic antibiotic choices, which may be considered for lactating patients with HS, include clindamycin, rifampicin, and intravenous ertapenem (Collier et al., 2021). Metronidazole is generally considered safe during pregnancy, but a recent labeling update by the U.S. Food and Drug Administration notes the inadequacy of current human data and possible risk of teratogenicity. Metronidazole has not been adequately studied among breastfeeding women (Collier et al., 2021).

**Fig. 4:**
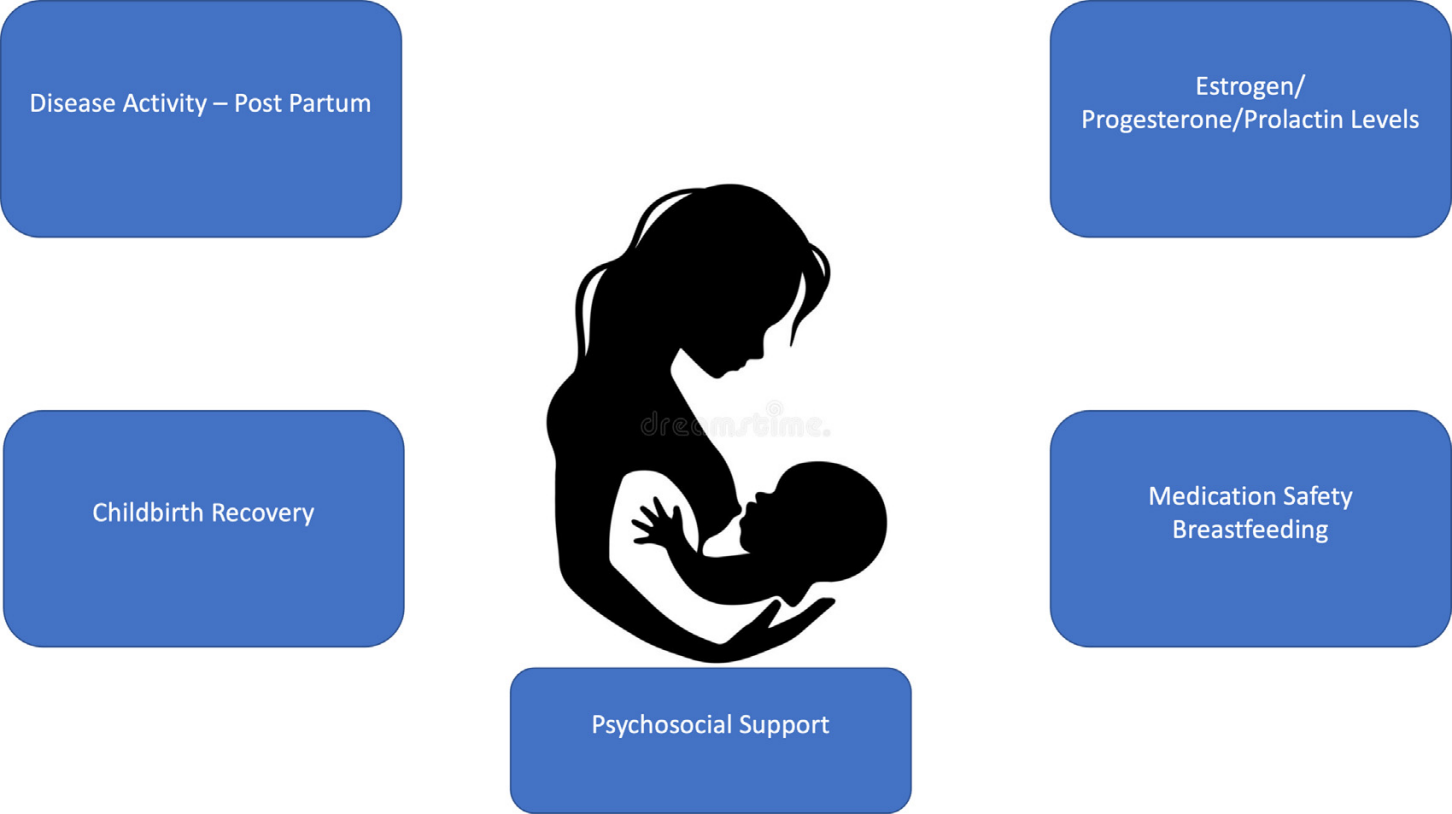
Breastfeeding and hidradenitis suppurativa: Management issues.

Important management goals and clinical considerations during the breastfeeding and postpartum period for women with HS include optimizing control of HS disease activity (considering the safety of medications depending on the breastfeeding preferences of the woman), aiding the mother to recover from childbirth, and providing psychosocial assistance to the mother and her support network (Fig. 4).

## Menopause and hidradenitis suppurativa

The impact of menopause on HS is unclear. Menopause has been reported to be associated with reduced HS disease severity in 48% of patients (Kromann et al., 2014). Evidence surrounding the effect of menopause on HS is overall, however, contradictory and inconsistent. Barth et al. (1996) reported that seven women surveyed continued to experience active HS disease after menopause. This concept is further supported by Fernandez et al. (2020), who found that 39.5% of their study group reported worsening of HS after menopause and 44.2% reported no change in their disease activity. The conflicting nature of the findings related to HS and menopause suggests that this is an area that requires further research in the future.

**Fig. 5:**
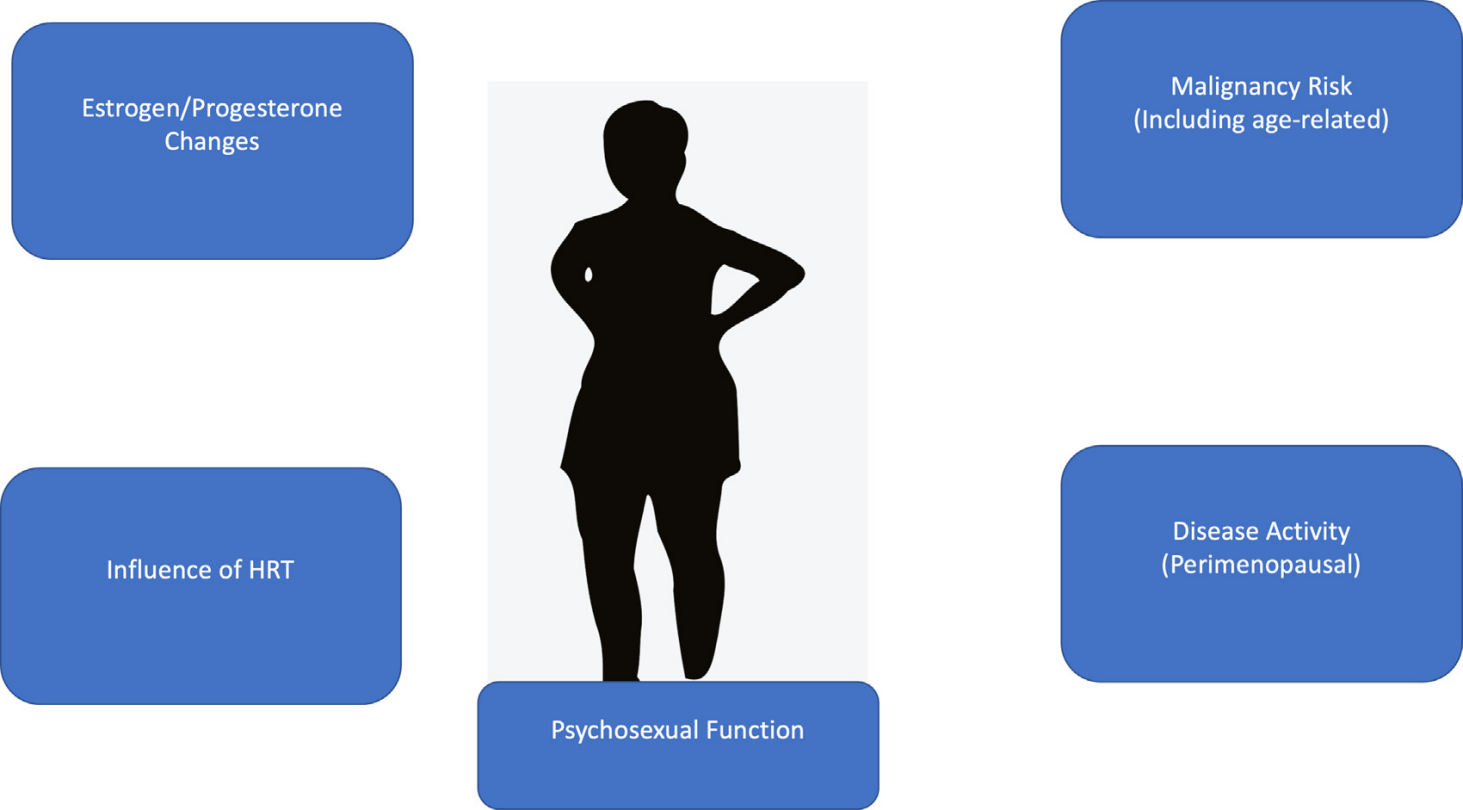
Menopause and hidradenitis suppurativa: Management issues.

Important management goals and clinical considerations during the perimenopausal period for women with HS include controlling any fluctuations in disease activity during the perimenopausal period, offering the woman psychosexual counseling (if appropriate), and monitoring for complications related to menopause, including the increased risk of malignancy (both disease- and age-related) and the influence of hormone replacement therapy on HS disease activity (Fig. 5). Individual recommendations regarding hormone replacement therapy need to be made based on the relationship between disease activity and menopausal symptoms, although anecdotally, hormone replacement therapy has a risk of worsening disease activity.

## Psychosocial impact of hidradenitis suppurativa

It is not surprising that a chronic, malodorous and painful condition such as HS is associated with a reduced quality of life secondary to physical, emotional, and psychological challenges (Kouris et al., 2016). Patients with HS often suffer from low self-esteem, poor sleep, sexual dysfunction, relationship dysfunction (including platonic and romantic), and poor mental health (Nguyen et al., 2020). In fact, patients with HS have been reported to be at a 2.42-times greater risk of suicide compared with the general population (Thorlacius et al., 2018).

One key challenge faced by patients with HS is sexual health and intimate relationships, a challenge that is reportedly more profound among women with HS (Thorlacius et al., 2018). This challenge is in part due to the anatomical location of HS lesions but is also secondary to pain, suppuration, fear of rejection, and a lower perceived physical attractiveness (Cuenca-Barrales, et al., 2020a). Sexual dysfunction is more common among women with HS (Cuenca-Barrales, et al., 2019), reported in up to 50% of women (Cuenca-Barrales et al, 2020b). These results are supported by Janse et al. (2017), who suggested that 59.7% of all patients with HS suffered from reduced sexual activity due to the impact of HS on their physical appearance (as reported by 89% of women), a diminished libido (91% of women), and the inconvenience caused by inflammatory disease (99% of women). Physical complications secondary to HS (e.g., pain) play a role in the intimate challenges experienced by patients, but there are also significant psychosocial aspects that are equally distressing to patients (Alavi et al., 2018; Jemec, 2018).

The social isolation often associated with HS has only been exacerbated during the coronavirus pandemic (Stout, 2020). One related study revealed that patients with HS responded positively to being involved in Zoom video communications and social media interactions as a way to improve on feelings of social isolation and share their experience living with HS with others (Stout, 2020).

## Current knowledge/management gaps and future directions

Although in many world regions, HS has been found to predominantly affect women, only very recently has the impact of HS on issues such as pregnancy, breastfeeding, menopause, and the psychosexual effect of the disease on women been explored in detail. A large knowledge gap is our limited understanding of the impact of HS across a patient's lifespan, which can be particularly devastating when HS begins during adolescence in young women. The social, psychosocial, physical, and financial effects of HS can be considered using a cumulative life course impairment model (Ibler and Jemec, 2020). HS is well-known to have a large negative impact on quality of life, but most published studies present a snapshot of the disease burden of HS in time rather than longitudinal data. For example, developing HS symptoms in private intertriginous areas, unpredictable malodorous suppurative drainage, and managing acute and chronic pain during teen years can lead to stigmatization and negative self-image. This can impact emerging sexuality and make developing personal relationships challenging.

Disease activity and treatments such as surgery can lead to significant intermittent absences during crucial educational years. The disruption to high school education can be enormous, altering postsecondary educational options and career choices, with an impact on financial security. Hence, the cumulative life course impairment of HS can occur through a number of mechanisms, and this should be evaluated in longitudinal studies of patients with HS. This could provide support for a more aggressive management approach earlier in the disease trajectory (Paek et al., 2017).

Several investigations are warranted to move this field forward, including mechanistic studies regarding how reproductive hormones impact disease activity, prospective registries to evaluate the efficacy and safety of HS treatments during pregnancy and pregnancy outcomes in patients with HS (Adelekun et al., 2020), implementation of effective targeted strategies to improve sexual function in women with HS, and longitudinal studies to explore the cumulative life course impairment that HS inflicts on patients. These studies would be part of a wider movement within the HS research community to understand the pathogenesis of the disease, expand the therapeutic armamentarium, and hopefully lead to the development of therapeutic and disease biomarkers in HS.

## Conclusion

When involved in the care of women with HS, we need to consider the unique challenges that these patients may experience, including menstruation, menopause, pregnancy, breastfeeding, and sexual dysfunction. To optimize patient care and quality of life, it is important that a multidisciplinary team is involved; this may include dermatologists, psychiatrists, psychologists, obstetricians, gynecologists, endocrinologists, and sexologists.

## Declaration of Competing Interest

Michelle A. Lowes has served on the advisory boards for Abbvie, InflaRx, Janssen, and Viela Bio, and consulted for Almirall, BSN Medical, Incyte, Janssen, Kymera, Phoenicis, and XBiotech. She is also on the medical board of the Hidradenitis Suppurativa Foundation. Jennifer L. Hsiao is on the board of directors for the Hidradenitis Suppurativa Foundation, a speaker for AbbVie, and a consultant for Novartis. John W. Frew has conducted advisory work for Janssen, Boehringer-Ingelheim, Pfizer, Kyowa Kirin, LEO Pharma, Regeneron, and UCB; participated in trials for UCB, Pfizer, and Eli Lilly; and received research support from Ortho Dermatologics.

## Appendix B Supplementary materials

Image, application 1
