# Supplementary material for: Clinical considerations in the management of hidradenitis suppurativa in women[image]
Source: Int J Womens Dermatol. 2021 Oct 29;7(5Part B):664–71. doi: 10.1016/j.ijwd.2021.10.012 (PMC8714605; doi:10.1016/j.ijwd.2021.10.012)
Supplement: Supplementary file 1 [file mmc1.pdf]

# HIDRADENITIS SUPPURATIVE IN WOMEN

Hidradenitis Suppurativa (HS) is a chronic inflammatory skin disease which disproportionately impacts women. The exact mechanisms by which hormones impact HS is not clearly understood. Women with HS face unique challenges with menstruation, hormonal fluctuation, sexual function, pregnancy, childbirth, and breastfeeding. Optimal management of HS in women relies upon an understanding of the hormonal influences in HS and available treatments.

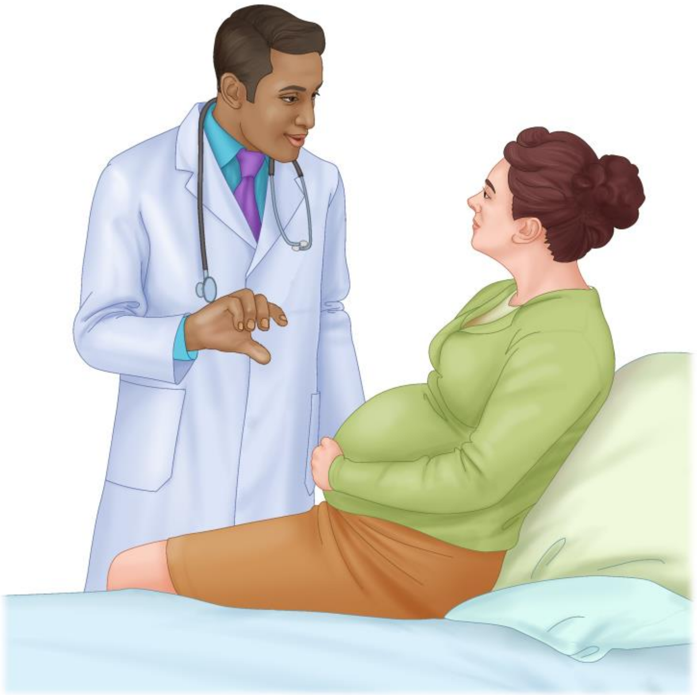

## Sex Hormones in Women can Influence Inflammation

Sex hormones such as estrogen, progesterone, and prolactin influence inflammation, yet the mechanisms in HS are unknown. Hormonal dysregulation is more prevalent in HS when associated with conditions such as polycystic ovarian syndrome, acne, hirsutism, and infertility. Estrogens and androgens drive inflammation through their action on specific immune cells, other cell types, and interactions with insulin.

## Impact of Menstrual Cycle and Pregnancy on HS

More than half of patients report a flare of their HS associated with their menstrual cycle suggesting that a rise in estrogens affects disease activity. Similarly, some women (20%) report a worsening of their HS during pregnancy, although a similar number (24%) report that their HS improved during pregnancy. The most common finding was a flare of HS after giving birth, suggesting that changes in hormone levels and prolactin may be associated with flares.

## Impact of Childbirth and Breastfeeding on HS

Since HS can impact childbirth, discussion with dermatologists and obstetrician/gynaecologists are necessary to provide an individual care plan. Monitoring for infection and other inflammatory complications are important during pregnancy and childbirth. HS can be an obstacle to breastfeeding due to pain and disease flares. Several therapies are safe in breastfeeding and ongoing psychosocial support is essential.

## Impact of the Menopause on HS

Whilst a large proportion of patients report an improvement in HS symptoms post-menopause, many patients find no change to their disease activity. The role of hormonally-based therapies in HS in the post-menopausal period need to be weighed against the background risk of malignancy (such as breast and ovarian carcinoma). Ongoing psychosocial and psychosexual support is needed during this period.

### FOR MORE INFORMATION

Kozera, EK, Lowes, MA, Hsiao, JL, Frew, JW. Clinical Considerations in the Management of Hidradenitis Suppurativa in Women. International Journal of Women's Dermatology 2021; 7(5): 518-525

**Authors:** John Frew, MD
**Conflict of Interest Disclosures:** None reported.
December 2021 | IJWD Patient Page
www.wdsijwd.org

The IJWD Patient Page is provided to the public from the International Journal of Women's Dermatology. The contents of the IJWD Patient Page including text, graphics, and images are provided for informational purposes only and should not be considered specific medical advice. Always consult a qualified medical professional for medical diagnosis, advice, and treatment. This page may be printed and photocopied for noncommercial use.
